# Role of α- and β-Synucleins in the Axonal Pathology of Parkinson’s Disease and Related Synucleinopathies

**DOI:** 10.3390/biom5021000

**Published:** 2015-05-19

**Authors:** Akio Sekigawa, Yoshiki Takamatsu, Kazunari Sekiyama, Makoto Hashimoto

**Affiliations:** Tokyo Metropolitan Institute of Medical Sciences, 2-1-6 Kamikitazawa, Setagaya-ku, Tokyo 156-0057, Japan; E-Mails: akio.sekigawa@zeiss.com (A.S.); takamatsu-ys@igakuken.or.jp (Y.T.); sekiyama-kz@igakuken.or.jp (K.S.)

**Keywords:** axonopathies, α-synucleinopathies, Parkinson’s disease (PD), dementia with Lewy bodies (DLB), transgenic (tg), globules

## Abstract

Axonal swellings are histological hallmarks of axonopathies in various types of disorders in the central nervous system, including neurodegenerative diseases. Given the pivotal role of axonopathies during the early phase of neurodegenerative process, axonal swellings may be good models which may provide some clues for early pathogenesis of α-synucleinopathies, including Parkinson’s disease and dementia with Lewy bodies (DLB). In this mini-review, such a possibility is discussed based on our recent studies as well as other accumulating studies. Consistent with the current view that dysfunction in the autophagy-lysosomal system may play a major role in the formation of axonal swellings, our studies showed globule, small axonal swellings, derived from transgenic mice expressing either human wild-type α-synuclein (αS-globule) or DLB-linked P123H β-synuclein (βS-globule), contained autophagosome-like membranes. However, other pathological features, such as abnormal mitochondria, enhanced oxidative stress and LRRK2 accumulation, were observed in the αS-globules, but not in the βS-globules. Collectively, it is predicted that αS and βS may be involved in axonopathies through similar but distinct mechanisms, and thus, contribute to diverse axonal pathologies. Further studies of the axonal swellings may lead to elucidating the pathogenic mechanism of early α-synucleinopathies and illuminating a strategy for a disease-modifying therapy against these devastating disorders.

## 1. Introduction

Since the discovery of a linkage of missense mutations in the α-synuclein (αS) gene to a rare familial dominant Parkinson’s disease (PD) in 1995 [1], numerous studies have established that aggregation of α-synuclein, a presynaptic protein with unknown functions, may play a central role in the pathogenesis of α-synucleinopathies [2]. These include PD, dementia with Lewy bodies (DLB), multiple system atrophy, neurodegeneration with brain iron accumulation type 1 (NBIA-1, formerly known as Hallervorden-Spatz disease), REM sleep behavior disorder, and Lewy body variants of Alzheimer’s disease (AD) [3,4,5]. Subsequently, dozens of risk factors for familial PD were identified, and their characterization has revealed that alterations of these molecules due to either gene mutations or environmental factors may lead to impairment of various pivotal cellular systems, such as the ubiquitin-proteasome system, autophagy-lysosomal system, and mitochondria, in the pathogenesis of both familial and sporadic PD [6]. More recently, genome wide association studies have shown that sporadic PD is associated with a variety of genes, including *SNCA* (a gene encoding αS), leucine-rich repeat kinase 2 (*LRRK2*), and *MAPT* (a gene encoding tau) [6]. Despite a wealth of information obtained from the molecular studies, there are currently no effective therapies for the α-synucleinopathies.

**Figure 1:**
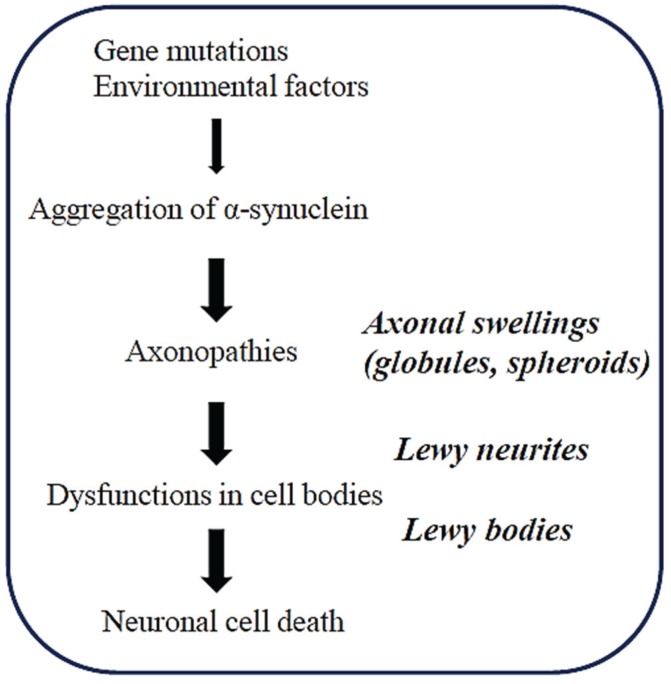
A hypothetical sequence of the pathogenesis of α-synucleinopathies. Axonopathies may precede the pathology of neuronal cell bodies in neurodegeneration. Various morphological changes (e.g., axonal swellings, Lewy neuritis and Lewy bodies) are associated with the progress of the disease.

Previous studies on the histopathology of the α-synucleinopathies have focused on Lewy bodies as hallmarks of the diseases. However, formation of Lewy bodies is a complicated process and the precise role of Lewy bodies in the pathogenesis of α-synucleinopathies is still unclear [7]. It is possible that it takes a long time for Lewy bodies to form in human brains [8]. This may be one reason why Lewy bodies are not found in the brains of rodent models of α-synucleinopathies whose life spans are much shorter when compared to that of humans [7]. Thus, it is likely that Lewy bodies are important for investigating the late pathogenesis of PD (Figure 1).

Recent clinical trials for AD suggest that therapeutic efficacy might be expected if the treatments were initiated earlier during the disease course [9]. Given the similar pathogenic mechanisms of neurodegeneration, this notion might also be true for PD and other neurodegenerative diseases. Considering that axonopathies may play a major part in the early pathogenesis of α-synucleinopathies (Figure 1), investigation of axonopathies may provide useful information on the early pathogenesis, leading to some clues for therapeutic strategies for α-synucleinopathies.

Axonopathies are accompanied by morphological alterations, including axonal swellings. Indeed, axonal swellings, such as globules and spheroids, have been associated with a number of diseases, including ischemia, trauma, neuroaxonal dystrophy, drug-induced axonopathies, and neurodegenerative disorders, as well as normal aging [10,11,12]. In particular, axonal swellings induced by chemical neurotoxicity are strikingly similar to those observed in amyotrophic lateral sclerosis [11,13]. Thus, it is possible that investigation of axonal swellings may provide valuable insights into the axonopathies in α-synucleinopathies.

## 2. Axonopathy is an Early Event in the Pathogenesis of Synucleinopathies

An increasing number of reports suggest that an axonal pathology may play a major role in the pathogenesis of neurodegenerative diseases [14,15]. In α-synucleinopathies, axonopathies caused by αS may play a critical role in the early neurodegeneration. In support of this notion, analyses of autopsied brains revealed that axonal pathology is widespread in various regions of the brains in the earliest stages of α-synucleinopathies [16,17]. Furthermore, the appearance of αS-positive Lewy neurites precedes that of Lewy bodies in both brains and cardiac sympathetic neurons [18]. In a similar manner, degeneration might begin in the distal axon and proceed towards the cell body in the brains of α-synucleinopathies [16,18].

Although the detailed mechanisms are still unclear, it is generally believed that aggregation of αS may play a central role in axonal pathology (Figure 1). It has been demonstrated that the neurotoxicity of αS is closely correlated with the levels of αS-containing protofibrils rather than with the levels of mature fibrils in various experimental models of α-synucleinopathies [2]. It is therefore possible that the toxic oligomeric forms of αS may target intracellular organelles and cellular pathways, including mitochondria, the ubiquitin-proteasome system, and the autophagy-lysosome pathway, leading to initiation of neurodegeneration in the axonal pathologies of α-synucleinopathies.

Accumulating evidence suggests that not only αS, but also two αS-related molecules, βS and γS, might be involved in the pathogenesis of axonopathies. In support of this theory, histological analyses of brains derived from α-synucleinopathies, such as DLB and NBIA-1, showed that all types of synuclein peptides are associated with neuritic pathology, such as that in both dystrophic neurites and spheroid structures [19,20]. Thus, elucidation of the mechanism by which all synuclein family members are involved in the process of axonal pathology may be important to understand the mechanism of axonopathies.

Axonal pathologies, such as axonal deposits of αS and axonal swellings, have been observed in various lines of transgenic (tg) mice expressing either wild-type- or mutated αS [21] Axonal pathology was also observed in tg mice expressing wild-type αS [22]. In addition, our microscopic studies have frequently observed globules, small axonal swellings, in the brain sections of tg mice expressing DLB-linked P123H β-synuclein (P123H βS-globules) [23] as well as in brains derived from tg mice expressing human wild-type αSyn (αS-globules) [24], in which Lewy body-like structures were rarely encountered. We hypothesized that axonal swellings formed in the brains of tg mice might be useful to investigate the axonal pathology caused by the synuclein family of peptides. We have shown that both αS and βS may be involved in the axonal pathologies of synucleinopathies through similar but distinct mechanisms.

## 3. Axonal Swellings in the Brains of a Tg Mouse Model with Synucleinopathies

### 3.1. Age-Dependent Formation of Globules in the Brains of a Tg Mouse Model of Synucleinopathies

Immunohistochemistry showed that α-synuclein-immunoreactive axonal swellings in the brains of αS tg mice were present in various areas, including the basal ganglia, thalamus, midbrain, and olfactory bulb [24]. These αS-globules were immunopositive for kinesin and were found in an age-dependent manner, with the higher numbers occurring in male mice compared to that in female mice. Since axonal dysfunctions may underlie the abnormal behaviors of tg mice, further studies may determine if globule formation precedes the appearance of behavioral changes, such as motor dysfunction and memory disturbance.

Both αS- and P123H βS-globules were immunopositive for γ-aminobutyric acid (GABA) and glutamic acid decarboxylase, suggesting that the globules might be derived from GABAergic neurons [23,24]. Although the mechanism through which globules are preferentially formed in GABAergic neurons is unclear, our results are consistent with previous studies showing that both dopaminergic neurons and other neuronal types, including large cholinergic inter-neurons and medium-sized GABAergic projection neurons, are involved in the neuritic pathology in the neostriatum of the PD brain [25,26].

### 3.2. Lysosomal Pathology is Commonly Observed in Both αS- and P123H βS-Globules

**Figure 2:**
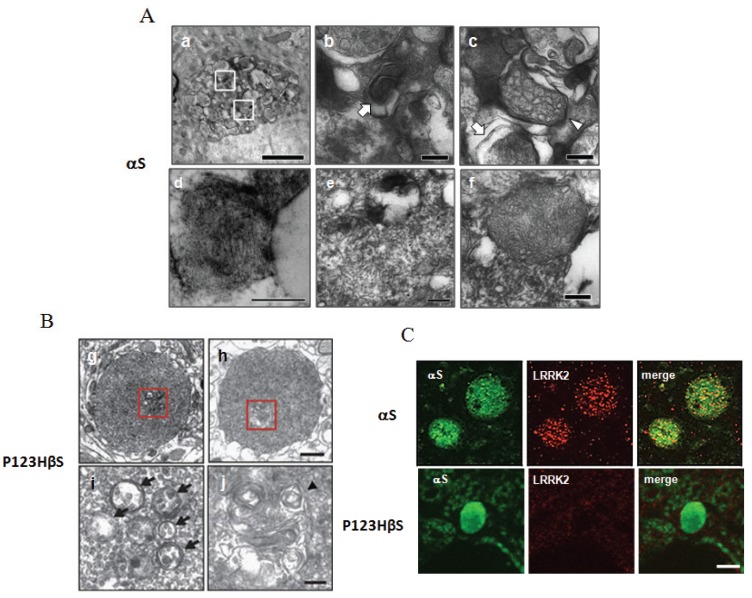
Histological analyses of the globules formed in the brains of both αS- and P123H βS tg mice. (**A**) Immunoelectron microscopy: The αS-globules (**a**) were characterized by lysosomal pathologies such as an αS-immunopositive multivesicular body (**b**: arrow) and autophagic vacuoles (**c**). (**b**) and (**c**) are magnifications from (**a**). Formation of curvilinear bodies (**d**) and a fingerprint profile (**e**) adjacent to a lipid droplet (**L**) are reminiscent of lysosome storage disease. Some mitochondria were swollen and deformed (**f**); (**B**) Immunoelectron microscopy: Abnormal lysosomal pathologies were also observed in the P123H βS-globules. (**i**) and (**j**) are magnifications from (**g**) and (**h**), respectively; (**C**) Immunofluorescence: Immunoreactivities of LRRK2 were detected in the αS-globules (upper 3 panels) but not in the P123H βS-globules (lower 3 panels). Reprinted from Mol. Brain, Sekigawa *et al.*, 5:34 with permission [25].

Immunoelectron microscopy showed both αS- and P123H βS-globules contained membranous elements, including autophagosome-like structures with double membranes, multivesicular bodies, and multilayered membranes, suggesting that the autophagy-lysosomal system may be impaired [24,27] (Figure 2A,B). Notably, some of the membranous structures were reminiscent of the fingerprint profile and curvilinear body that are frequently associated with lysosomal storage diseases, such as neuronal ceroid-lipofuscinosis and gangliosidosis [28].

In addition, immunofluorescence studies showed that the globules were immunopositive for various gangliosides, including GD1a, GD3, GM1, GM2, and GM3 [24,29]. Based on our previous reports regarding the protective effects of gangliosides on lysosomal pathology in rat B103 neuroblastoma cells expressing P123H βS [30,31], we speculate that gangliosides might play some protective roles in axonal pathology. Finally, lysosomal activities in brains derived from both αS- and P123H βS-tg mice were significantly decreased compared with that of non-tg littermates [23,24], suggesting that autophagosome-like membranes might have accumulated due to decreased clearance by lysosomes.

A recent study suggested that dysfunction of the autophagy-lysosome pathway may be one of the major contributors to axonal swellings [32]. Failure to degrade either subcellular materials or organelles at distal axons and/or at nerve terminals or failure to export these materials by axonal transport has been shown to produce swollen nerve terminals [33,34]. Such mechanisms might be involved in the formation of αS- and P123H βS-globules. Together, these results suggest that down-regulation of the lysosome pathway may be a common mechanism of axonal pathology in both αS- and P123H βS-tg mice.

### 3.3. Alterations of Mitochondria in the αS-Globules but Not in the P123H βS-Globules

In addition to lysosomal pathology, immunoelectron microscopy showed accumulation of mitochondria in the αS-globules but not in the P123H βS-globules [24]. Some αS-globules displayed clustering of mitochondria, while others had swollen mitochondria in the peripheral regions of the globules (Figure 2A). Consistent with the deformation of the mitochondria, there was a clear decrease in the osmophility, suggesting increased pH in the mitochondrial intermembrane space [35]. However, more severe mitochondrial pathologies, such as distorted and vacuolated mitochondria, were not observed.

An immunofluorescence study showed that αS-globules were immunopositive for various mitochondrial markers, including VDAC1, cytochrome C, and heat shock protein 60 (HSP 60) [24]. The diffuse pattern of VDAC1 staining suggested possible damage of the mitochondrial outer membrane. However, it is unlikely that apoptosis was involved since cytochrome C and HSP 60 staining still localized in the mitochondria. Consistent with these results, it was previously described that abnormal mitochondria accumulated in the axons in tg mice expressing prion promoter-driven αS [21].

### 3.4. Oxidative Stress Conditions are Enhanced in the αS-Globules and to a Lesser Extent in the P123H βS-Globules

Abnormal accumulation of mitochondria in the αS-globules might stimulate oxidative stress [24]. Consistent with this notion, αS-globules were associated with oxidative stress, as assessed by staining for 4-hydroxy-2-nonenal (4-HNE), a product of biological lipid peroxidation and nitrated αS. Phosphorylation of αS was evaluated as another possible posttranslational modification. The αS-globules were modestly stained with anti-phospho-αS (phospho-Ser129) antibody, indicating that phosphorylation of αS may not be critical for globule formation.

In contrast to the αS-globules, mitochondria were rarely observed in the P123H βS-globules [23]. In accord with the absence of mitochondria, oxidative stress conditions in the P123H βS-globules, as assessed by anti-4HNE antibody, was significantly weaker than that in the αS-globules. While phosphorylation of endogenous mouse αS in the P123H βS-globules was similar to that in the αS-globules. The mechanism through which P123H βS causes oxidative stress without mitochondria is unclear, but it is noteworthy that cholesterol staining was positive in the P123H βS-globules but not in the αS-globules. Given that cholesterol and its metabolites are implicated in reducing oxidative stress in the pathogenesis of neurodegenerative diseases [36], cholesterol translocation could be partly due to the accumulation of P123H βS in the globules. A further study is warranted to test this intriguing possibility.

### 3.5. Accumulation of LRRK2 in the αS-Globules but Not in the P123H βS-Globules

Notably, immunofluorescence studies frequently detected LRRK2 (PARK8) in the αS-globules (Figure 2C) [24]. The staining exhibited a small granular pattern, suggesting that LRRK2 might localize at the membranous structures. Similar to the case of abnormal mitochondria, LRRK2 immunoreactivity was detected in the αS-globules but not in the P123H βS-globules [23,24].

LRRK2 may be actively involved in the axonal pathology. Indeed, it was previously shown that LRRK2 was crucial for regulation of both neurite formation and length [37]. Knockdown of LRRK2 led to long, highly branched neuritic processes, whereas constructs with increased kinase activity exhibited short simple processes in neuronal cultures (or transduced nigrostriatal models) [37]. More recently, LRRK2R1441G BAC tg mice were shown to have various characteristic axonal pathologies, including large tyrosine hydroxylase-positive spheroid-like structures, dystrophic neurites, and enlarged axonal endings.

The specific accumulation of LRRK2 in the αS-globules naturally leads to the speculation that LRRK2 may cooperate with αS in axonal pathology [24]. In support of this possibility, both αS and LRRK2 have been shown to be commonly involved in pathologies, such as impairment of cytoskeletal dynamics and dysregulation of the protein degradation system [38]. Moreover, it was recently shown that various neuropathological features of A53T αS tg mice, such as impaired microtubule dynamics, Golgi disorganization, and decreased proteasomal activity, worsened with cross-breeding with LRRK2 tg mice, but improved with genetic ablation of LRRK2 [39]. Further investigation is required to determine whether αS and LRRK2 cooperate with each other to produce diverse pathologies, including axonal degeneration.

Since many familial PD risk genes have been implicated in disorders of subcellular organelles, such as lysosomes and mitochondria [6], we examined whether any of these factors were involved in globule formation in both αS tg mice and P123H βS tg mice. Although it has been well documented that Parkin (PARK2) and PINK1 (PARK6) are autosomal recessive factors that are involved in the maintenance of mitochondrial quality, and that mutations in these genes are causative for mitophagy [40], neither Parkin nor PINK1 was immunopositive in the αS- and P123H βS-globules. In addition, there was no immunoreactivity for DJ-1 (PARK7) in both types of globules.

## 4. Axonal Protection Strategy Based on βS Actions

Given that βS is an inhibitor of αS aggregation [41,42,43], one may naturally wonder if neurodegenerative conditions in axonal pathology may be exacerbated by αS, while they are negatively regulated by βS. Since several studies have indicated decreased βS expression in some brain regions in α-synucleinopathies, compensation for decreased expression of βS may be an effective strategy aimed at axonal protection [44]. For instance, it was previously shown that viral delivery of βS to the brains of αS tg mice decreased αS aggregation [45].

It should be noted that full-length βS might not always be protective. βS may become pathogenic through gene mutations (e.g., P123H or V70M) [46]. Furthermore, it was recently shown that adeno-associated virus-mediated overexpression of wild type of βS resulted in the formation of proteinase K-resistant aggregates of βS in primary cultured neurons and in dopaminergic neurons in rat brain [47]. In this study, amyloid channels on the membranes were formed by βS, accompanied by a mild mitochondrial pathology. Indeed, a previous report using an *in vitro* cell-free system detected amyloid fibrils of wild type βS in the presence of specific metal ions and glycosaminoglycan macromolecular crowding agents [48]. Consistent with these results, our studies suggest that βS may be involved in lysosomal dysfunctions, although βS appears not to be involved in the cause of abnormal mitochondria in axonal pathology. Collectively, it is likely that βS may possess gain-of-function properties in neurodegeneration.

**Figure 3:**
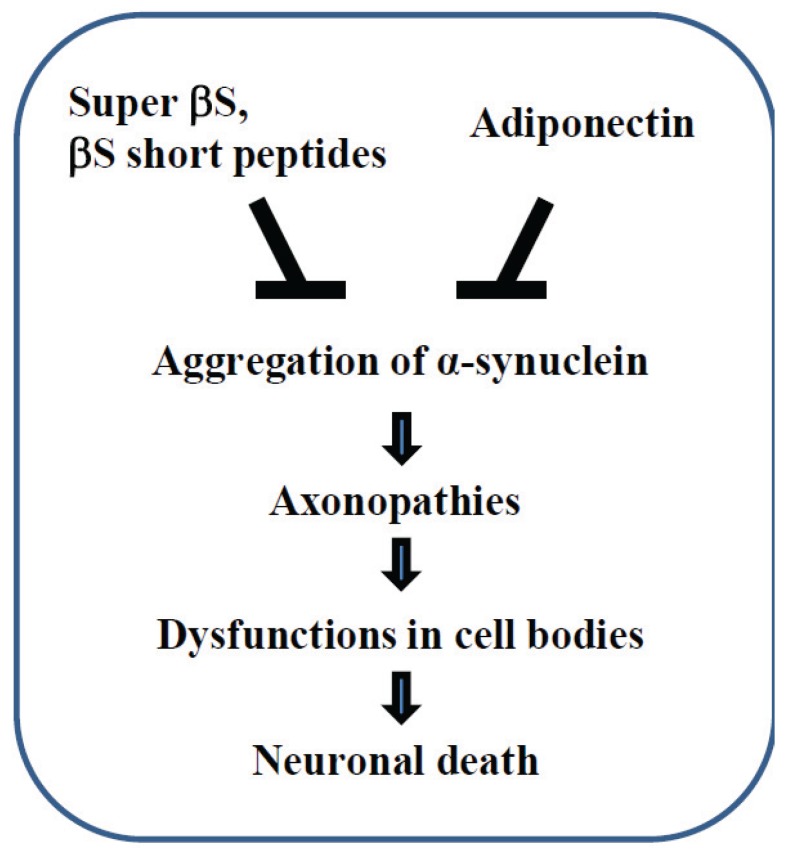
Therapeutic strategy against axonal pathology of α-synucleinopathies. Modified forms of βS, such as “super” βS and “short peptides” derived from βS; please see the main text for details, may be used to suppress the aggregation of αS. Adiponectin may be also effective in protecting against axonal degeneration.

If the aggregation properties of full-length βS are problematic from a therapeutic point of view, one potential strategy may be to use “short peptides” derived from βS [44] (Figure 3). Indeed, this strategy was effective for prevention of αS aggregation in the brain of αS tg mice [49]. More recently, it was shown that locomotor activity and accumulation of A53T αS in the brain of a Drosophila model of PD expressing A53T αS was significantly ameliorated when the flies were fed with retro-inverso βS peptides [50]. Alternatively, engineering of a “super” βS that is resistant to patho genic structural conformational changes, compared with wild-type βS may be an interesting strategy [44] (Figure 3).

Furthermore, there might be other therapeutic strategies for α-synucleinopathies which are effective in removing the aggregated pathological βS. In this regard, adiponectin may be a good candidate because this molecule decreases expression of aggregated αS in cells and in a mouse model of α-synucleinopathies [51] (Figure 3).

## 5. Conclusions

While there has been extensive research using Lewy bodies to examine the histopathology of α-synucleinopathies [8,52], axonal swellings have been relatively neglected in this field. One reason is that such morphological alterations have been described in other types of diseases [13,53] as well as in normal aging conditions [54]. It is possible that axonal swellings might be formed in response to various neurotoxic stimuli, including amyloid protofibrils. Thus, axonal swellings are apparently not specific to α-synucleinopathies, however, they might be good models of early axonal pathology in α-synucleinopathies. In support of this possibility, our analyses of the globules formed in the brains of tg mouse models of synucleinopathies suggest that both αS and βS may be involved in axonopathies through similar but distinct mechanisms. We suggest that a better understanding of the role of the synuclein family of peptides in axonal pathology is critical for the protection of axon and for the development of therapeutics agents for α-synucleinopathies.
